# Interventions to tackle health inequalities in cardiovascular risks for socioeconomically disadvantaged populations: a rapid review

**DOI:** 10.1093/bmb/ldad025

**Published:** 2023-09-18

**Authors:** Yu Fu, Ge Yu, Naswa Maulana, Katie Thomson

**Affiliations:** Department of Primary Care & Mental Health, Institute of Population Health, University of Liverpool, 1-3 Brownlow Street, Liverpool, L69 3GL, UK; King’s Health Economics, Health Services and Population Research, Department of Psychiatry, Psychology & Neuroscience, King’s College London, David Goldberg Centre 18 De Crespigny Park, London, SE5 8AF, UK; Population Health Sciences Institute, Faculty of Medical Sciences Newcastle University, Baddiley-Clark Building, Richardson Road, Newcastle upon Tyne NE2 4AX, UK; Population Health Sciences Institute, Faculty of Medical Sciences Newcastle University, Baddiley-Clark Building, Richardson Road, Newcastle upon Tyne NE2 4AX, UK; National Institute for Health Research Applied Research Collaboration, North East and North Cumbria, Cumbria, Northumberland, Tyne & Wear NHS Foundation Trust St Nicholas Hospital Gosforth, Newcastle Upon Tyne NE3 3XT, UK

**Keywords:** lipid management, socioeconomically disadvantaged, cardiovascular disease, health inequalities, rapid review

## Abstract

**Introduction:**

Cardiovascular disease (CVD) has shown significant health inequalities for people with low socioeconomic status associated with more risk factors. This review was to synthesize interventions that targeted CVD risks and outcomes among socioeconomically disadvantaged populations and to understand the impact associated with these interventions.

**Sources of data:**

Cochrane CENTRAL, MEDLINE, Embase, PsycINFO and CINAHL were searched for records published in the last decade using a systematic search strategy, complemented by screening the reference lists and citation indexes. Nineteen studies were included and a narrative synthesis with the effect direction plot was undertaken in which studies, interventions, participants and outcomes were examined according to the intervention type focusing on behaviours, lifestyle, education, medication and monitoring.

**Areas of agreement:**

No universal definition of disadvantaged socioeconomic status was used with common factors relating to racial/ethnic minorities, low income and low or no health insurance. Mixed effects of interventions were reported on clinical outcomes including weight, body mass index, blood pressure, glycated haemoglobin and cholesterol.

**Areas of controversy:**

Inconsistent effect was reported due to a large variety of settings, participants and intervention components although they are considered necessary to address the complex health needs of socioeconomically disadvantaged populations.

**Growing points:**

There is inadequate evidence to determine whether any of the intervention types are effective in optimising lipids management for socioeconomically disadvantaged populations.

**Areas timely for developing research:**

Research is needed with mixed evidence using real world evaluation and lived experience combined with health economic evaluation, on both mental and physical health outcomes.

## Introduction

Hyperlipidaemia is characterized by elevated levels of lipids caused by acquired and genetic disorders. It is a chronic progressive disease associated with the development of cardiovascular disease (CVD), a leading cause of mortality resulting in nearly 18 million deaths annually, representing 32% of all deaths worldwide.^1^ CVD is caused by thrombosis or atherosclerosis restricting blood flow and is commonly presented as coronary heart disease (including angina and myocardial infarction), stroke, transient ischaemic attack and peripheral arterial disease. It is estimated that CVD could cost £9 billion in healthcare per year.^2^

CVD risk can be reduced by modifying blood lipid profile targeting total cholesterol, non high density lipoprotein cholesterol and triglyceride level. Both national and international guidelines recommend assessment and management strategies including blood tests, statin treatment, modification of other risk factors such as smoking and obesity, management of secondary causes of dyslipidaemia and outcomes monitoring.^3^ Evidence also supports the effectiveness of lipid lowering therapies^4^ and lifestyle modifications^5^^,^^6^ in preventing CVD in adults. Consequently, there has been a decrease in overall CVD incidence over the last three decades with a stable mortality-to-incidence ratio worldwide.^7^

Whilst the National Health Service (NHS) Long Term Plan has set up CVD ambitions for the next 10 years targeting atrial fibrillation, blood pressure and cholesterol,^8^ CVD has shown significant health inequalities for people with low socioeconomic status associated with less access to care and more risk factors. People in the most deprived areas in UK were four times more likely to die prematurely due to CVD than those in the most affluent areas from 2017 to 2019.^9^ Also, high blood pressure is 30% more likely to be detected in the most deprived areas which presents the biggest single risk factor for heart attack and stroke.^9^ This could be attributed to a range of biological, behavioural and psychosocial risk factors that are more prevalent in disadvantaged individuals.^10^ The COVID-19 pandemic has further amplified the problems experienced as lockdowns, quarantines, and closure of some supporting services have all disrupted care and exacerbated health inequalities in CVD. This may result in a further considerable increase in CVD incidence, particularly with acute pathologies such as stroke, acute coronary syndrome and cardiogenic shock among individuals with lower socioeconomic status and vulnerable elderly populations.^11^

Interventions should target specific risk factors associated with low socioeconomic status when aiming to improve health outcomes. However most trials and evidence have not been adequately powered to engage people with low socioeconomic status in detecting effects^5^ in improving CVD events,^12^ mortality,^13^ hypertension,^14^ diabetes incidence,^15^ metabolic syndrome,^16^ diet^17^ and physical activity^18^^,^^19^ as well as reporting intervention harms.^20^^,^^21^ As such, there is a limited evidence base for interventions targeting socioeconomic disadvantage. In practice, the NHS Health Check launched in 2009 was designed to enable early detection of stroke, kidney disease, heart disease, type 2 diabetes or dementia amongst adults in UK aged 40 to 74.^22^ However there has been poor engagement reported with the most disadvantaged groups with a higher risk of developing CVD.^23^ This led to a call for action to increase awareness and uptake from Public Health England in 2014, yet data suggests that less than half of the socioeconomically disadvantaged populations attended and received follow up support.^24^ Implications for both research and practice highlight that there is a need to investigate optimised interventions tailored for the characteristics and needs of individuals with low socioeconomic status. With limited but emerging research set up tackling health inequalities, there is no synthesis of current literature of interventions targeting CVD risks and outcomes for socioeconomically disadvantaged populations, hence this review.

The aim of this review was, therefore, to synthesize interventions that targeted CVD risks and outcomes among socioeconomically disadvantaged populations and to understand the impact associated with these interventions.

## Methods

This review was undertaken and reported following the Cochrane Rapid Reviews Guidance^25^ and the Preferred Reporting Items for Systematic Reviews and Meta-Analyses (PRISMA) statement.^26^ It has been registered with PROSPERO (registration number CRD42022348881). The protocol has been published^27^ elsewhere.

### Eligibility

#### Type of studies

This review focused on empirical studies published in peer-reviewed scientific journals, within the last 10 years (to mirror the NHS long term plan) and in the English language. To ensure a degree of commonality in the health system as well as socioeconomic and demographic content, studies were included only if they were conducted in Organization for Economic Co-operation and Development (OECD) countries.^28^

#### Type of participants

Studied were included if they involved adults with common CVD comorbidities who were from disadvantaged socioeconomic backgrounds (income, education, social class, deprivation, poverty or an area-based proxy for deprivation derived from place of residence). Comorbidities were referred to as conditions that can increase the risk of developing CVD including hypertension, diabetes mellitus, chronic kidney disease (CKD) and dyslipidaemia.^29^

#### Type of interventions

Multifaceted interventions were included due to the need for the intervention to improve multiple factors associated with low socioeconomic status.

#### Type of outcome measures

There is no universal recommendation for the core outcomes sets in studies on CVD prevention,^30^^,^^31^ studies were included regardless of outcomes measured or reported for health outcomes. This may include vascular related outcomes, cognitive and functional outcomes, lifestyle, medical risk factors, cardioprotective medications and patient reported outcome measures. Any measures of professionals’, patients’ and/or families’ knowledge, attitudes or satisfaction were also included.

### Data sources and search strategy

Detailed search strategies for Cochrane CENTRAL, MEDLINE, Embase, PsycINFO and CINAHL were developed by YF refined by NM and validated by an information specialist. Boolean operators were also used to maximize the retrieval of relevant records (supplemental material 1). The searches were conducted on May 26, 2022.

Identified citations were exported to Endnote v20 for initial deduplication before being exported to Rayyan for title and abstract screening. This was conducted by NM and a random 10% of citations were independently screened by YF and KT. Full texts were retrieved and screened where citations appeared to meet the eligibility or where a decision to exclude could not be made on the information provided. Reference lists and citation indexes of relevant articles were scrutinized. Any discrepancies were resolved by discussion amongst the research team.

### Data extraction

A data extraction sheet was developed and further piloted with two retrieved studies including the author’s last name, publication date, location and setting, study design, the aim of the study, a brief description of the intervention, inclusion and exclusion criteria, method of recruitment, outcome measures, participant characteristics (number, gender, age and ethnicity) and primary findings. Where a study appears to have multiple citations, all information from multiple citations was used.

### Quality assessment

Quality appraisal of included studies was performed using modified versions of the Critical Appraisal Skills Programme (CASP) tool. Any discrepancies were resolved by discussion amongst the research team. The decision was made to include all papers in data extraction irrespective of methodological quality to provide a complete overview of the existing literature.

### Data analysis

Due to the level of heterogeneity of study settings, participants, intervention components and outcomes reported, a narrative synthesis was undertaken by YF, GY and NW, validated by KT to focus on the intervention components, reported effects and mechanisms leading to the outcomes. Interventions and outcomes were grouped according to the design and elements of the intervention and the effect size and 95% confidence interval reported. The effect direction plot table was made to support the synthesis and visualization of effect direction data according to the outcomes reported across the included studies indicating the impact on health outcomes, together with shades to represent study quality.^32^

## Results

### Study selection

**Fig. 1:**
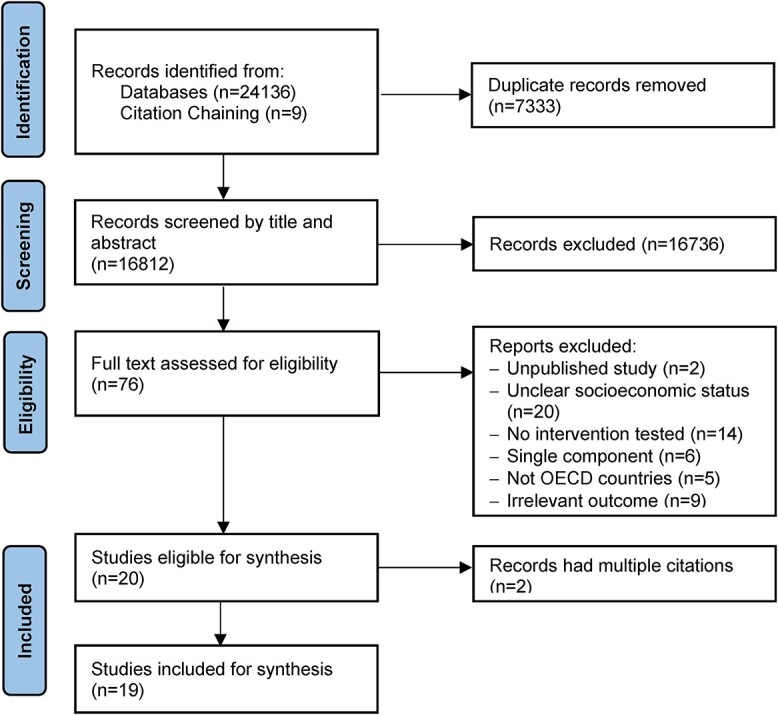
PRISMA flowchart.

A total of 24 136 records were yielded from the initial search and nine records were further identified from citation chaining. This resulted in 16 812 records after 7333 duplicates were removed. Following the screening of titles and abstracts, 76 studies were retrieved in full text and 56 were further excluded with common exclusion mainly due to lack of description of disadvantaged socioeconomic status (*n* = 20), intervention (*n* = 15) or outcomes (*n* = 9). A total of 20 citations were included with two^33^^,^^34^ reporting the same study (Fig. 1).

### Study characteristics

The majority of studies included were conducted in the US,^33–49^ and the rest were in the UK,^50^ Italy,^51^ Mexico and Honduras^52^ with participants ranging from 18 to 1665 adults with disadvantaged socioeconomic status. Participants had mixed ethnicity comprising Hispanic and Latino Americans dominated,^36^^,^^37^^,^^40^^,^^41^^,^^43^^,^^44^^,^^47^^,^^49^ Black dominated^33^^,^^35^^,^^42^^,^^45^^,^^48^ and White dominated^38^^,^^39^^,^^46^^,^^50^ in all studies that reported ethnicity. The particpants were recruited from communities,^35^^,^^37^^,^^39^^,^^40^^,^^44^^,^^47^^,^^49^ primary care,^33^^,^^45^^,^^46^^,^^48^ free clinics,^36^^,^^38^ hospitals,^41^^,^^51^ general practices,^50^ senior centre and residential facilities,^42^ family health centres,^43^ and a combination of private and public clinics and primary care practice and community outreach.^52^ Over half of the participants were female (51.6 to 100%) in all included studies which reported gender.

Of the included studies, 13 were randomized controlled trial (RCTs) with a follow-up period ranging from 6 weeks to 24 months and 6 were cohort studies from 4 to 12 months. Interventions investigated included behavioural interventions,^35^^,^^47^^,^^48^^,^^50^ lifestyle interventions,^33^^,^^36^^,^^37^^,^^39^^,^^40^^,^^43^^,^^44^ education based interventions,^38^^,^^42^^,^^45^^,^^49^^,^^51^ medication based interventions^41^ and monitoring based interventions.^46^^,^^52^ Amongst 13 RCTs, 8 compared with the usual care and the rest were screening and educational handouts,^39^^,^^40^ monitoring and coaching^41^ and information provision.^50^^,^^52^

Included studies focused on either single or multiple conditions including CVD risk factors,^37–40^^,^^42^^,^^51^ diabetes,^38^^,^^45–47^^,^^49^ hypertension,^35^^,^^38^^,^^41^^,^^52^ obesity,^33^^,^^43^^,^^44^^,^^50^ metabolic syndrome^36^ and CKD.^48^ No universal definition was used for disadvantaged socioeconomic status, participants were mainly judged by racial/ethnic minority,^33^^,^^35–37^^,^^41^^,^^44^^,^^45^^,^^47^^,^^48^ low income,^33^^,^^37^^,^^38^^,^^41^^,^^43–45^^,^^49^^,^^50^ low or no health insurance,^36^^,^^38–40^^,^^49^^,^^52^ locations,^43^^,^^46^^,^^50^^,^^51^ substance abuse and homelessness.^42^

Common outcomes reported include changes in weight,^33^^,^^35–37^^,^^42–45^^,^^49^^,^^50^ BMI,^35–37^^,^^39^^,^^40^^,^^43^^,^^44^^,^^49–51^ systolic blood pressure (SBP),^33^^,^^35–42^^,^^44–52^ diastolic blood pressure (DBP),^33^^,^^35^^,^^37–42^^,^^44–47^^,^^49–52^ glycated haemoglobin (HbA1C),^38^^,^^43^^,^^45–47^^,^^49^ total cholesterol,^33^^,^^38–40^^,^^42^^,^^44^^,^^47^ low-density lipoprotein (LDL) cholesterol^33^^,^^36^^,^^38–40^^,^^44–47^^,^^51^ and high-density lipoprotein (HDL) cholesterol.^33^^,^^38^^,^^44^^,^^47^^,^^51^ None of the studies measured mental health wellbeing. The characteristics of the included studies are presented in Table 1.

**Table 1:** Characteristics of included studies (*n* = 19)

**Citation**	**Country; setting; SES definition**	**Design; intervention length; Follow up**	**Components of intervention (I) vs Control (C)**	**No. analysed; Female%; Age (mean,SD)**	**Race/ethnicity**	**Primary outcome (PO); secondary outcome (SO)**
35	US; Community health centres; A predominately racial/ethnic minority patient population	RCT; 24 months	Behavioural intervention: —weight loss —hypertension self-management vs Usual care	314; 68.5%; I: 54.58 ± 10.77 C: 54.67 ± 11.03	71.2% non Hispanic Black 3.6% non Hispanic white—13.2% Hispanic—1.6% American Indian—0.5% Asian—0.5% Hawaiian/pacific islander—8.5% > 1 race—0.8% unknown race	PO: Change in body weight (kg) SO: change in systolic blood pressure (mm mercury)
36	US; Free clinic and local churches; A predominantly Hispanic cohort of low-income, uninsured individuals	Cohort; 8 weeks; 12 month	Lifestyle intervention —metabolic syndrome screening —linguistically-appropriate educational materials	126; 73.0%; mean: 49.8	Majority was Hispanic	No PO or SO defined. Outcomes were described as ‘percent stable or improved’ and ‘percent and total amount changed’, for each category
37	US; Community health fairs; A greater proportion of Mexican-American residents (almost 95%), a lower socio-economic status and less access to health care compared to the rest of El Paso	Cohort; 4 months	Lifestyle intervention: —physical activity —dietary intake —heart-healthy education	413; 86.0%; 46.6 ± 12.8	Hispanic	No PO or SO defined. Participants completed clinical measurements including height (inches), weight (pounds), waist circumference (inches), hip circumference (inches), and BP (mm Hg). A sum score of CVD risk factors including screening practices, presence of chronic conditions, and health behaviours was calculated.

(*Continued*)

**Table 1:** Continued

**Citation**	**Country; setting; SES definition**	**Design; intervention length; Follow up**	**Components of intervention (I) vs Control (C)**	**No. analysed; Female%; Age (mean,SD)**	**Race/ethnicity**	**Primary outcome (PO); secondary outcome (SO)**
38	US; Rural primary care free clinic; Individuals aged 18–64 years who are uninsured, live in 1 of the 6 surrounding counties, and have a household income 150% or more below the federal poverty level	Cohort; 12 months	Education focused: —vitals and medication history assessment —30 min appointment with pharmacist for disease state and medication education and medication therapy adherence —30 mins appointment with health coach for dietary and physical activity recommendations, goal setting —follow up appointments as mandated	95; 51.6%; 49.6 ± 9.97	83.2% white; 16.8% African American	PO: HbA1C, SBP, DBP and total CHL, LDL, HDL, and triglycerides SO: ED visits and hospital admissions
33	US; Primary care clinics; Racially diverse, low-income populations across Louisiana	Cluster RCT; 24 months	Lifestyle intervention —education sessions —portion-controlled foods vs Usual care	803; 84.4%; 49.4 ± 13.1	67.3% Black; 25.9% White; 6.8% Other	PO: mean per cent weight loss from baseline to month 24 SO: cardiometabolic risk factors
40	US Community; Underinsured or uninsured woman aged 40–64 years	RCT; 12 week; 12 months	Lifestyle intervention: —CVD risk factor screening; —CVD related educational handouts —referrals as needed—follow up assessment at 12 and 24 months —postcards and newsletters —life change intervention (nurtition, physical activity) vs —CVD risk factor screening; —CVD related educational handouts —referrals as needed —follow up assessment at 12 and 24 months —postcards and newsletters	180; 100% 50.87 ± 6.88	Hispanic	PO: FAFQ fat summary scale score; the FAFQ fibre summary scale score; the FVS score (total intake of fruit and vegetable servings per day); the CHAMPS all intensity physical activity; the CHAMPS moderate intensity physical activity; and clinical measures of total CHL, total glucose, LDL and BMI. No SO defined.

(*Continued*)

**Table 1:** Continued

**Citation**	**Country; setting; SES definition**	**Design; intervention length; Follow up**	**Components of intervention (I) vs Control (C)**	**No. analysed; Female%; Age (mean,SD)**	**Race/ethnicity**	**Primary outcome (PO); secondary outcome (SO)**
39	US Community; Underinsured or uninsured woman aged 40–64 years	RCT; 12 week; 12 months	Lifestyle intervention:—CVD risk factor screening; —CVD related educational handouts —referrals as needed —follow up assessment at 12 and 24 months —postcards and newsletters —life change intervention (nurtition, physical activity) vs —CVD risk factor screening; —CVD related educational handouts —referrals as needed —follow up assessment at 12 and 24 months —postcards and newsletters	833; 100%; 52.5 ± 7.0	84.2% non-Hispanic white; 1.7% Hispanic; 6.7% African; American/Asian/others 7.4% unknown	PO: dietary (FAFQ and the total number of FVS), physical activity (mean total hours and moderate hours per week), and clinical outcomes (SBP, DBP, total blood CHL, LDL, blood glucose, and BMI). No SO defined.
41	US; Public hospital; Low-income, largely minority patients	RCT; 6 months	Medication focused: —home blood pressure monitoring —weekly health coaching —home titration of blood pressure medications vs '— home blood pressure monitoring —weekly health coaching	204; 63.2%; 60.4 ± 12.1	8.3% non Hispanic white; 10.8% black/African American; 45.6% Latino; 34.8% Asian	PO: change in SBP SO: change in DBP, percent of patients < 130/80 mmHg for diabetic patients and < 140/90 mmHg for nondiabetic, primary care visit frequency.
50	UK; General practices; London boroughs of Tower Hamlets and Hackney, both areas with high levels of social deprivation	RCT; 8 weeks; 12 months	Behavioural intervention: —weekly group sessions on standard cognitive behavioural interventions, dietary advice, self-monitoring —monthly maintenance sessions —information on local exercise provision —information on orlistat vs —information on local exercise provision—information on orlistat	291; 71.5%; I: mean 47 C: mean 45	39.7% White British; 11.5% white other; 23.9% black; 13.0% Asian; 3.6% mixed; 7.0% other	PO: weight change SO: change in BMI, waist circumference, BP, and proportion of participants losing at least 5% and 10% of baseline body weight

(*Continued*)

**Table 1:** Continued

**Citation**	**Country; setting; SES definition**	**Design; intervention length; Follow up**	**Components of intervention (I) vs Control (C)**	**No. analysed; Female%; Age (mean,SD)**	**Race/ethnicity**	**Primary outcome (PO); secondary outcome (SO)**
42	US; Senior centre and residential facility; Women with histories of substance abuse and homelessness	Cohort; 6 months	Education focused: —screenings —coaching sessions —teachings materials —an LS7 health guide	18; 94.4%; Senior: 71 ± 3.1 Residential: 53 ± 5.9	Mostly African American, others were Hispanic and caucasian	No PO or SO defined. Below are reported:—SBP, DBP—blood glucose—blood CHL—weight—My Life Check score
43	US; Family health center; Lawrence, Massachusetts, a primarily lowincome, 60% Latino city	RCT; 12 months	Lifestyle intervention: —providing information —promoting positive attitudes —building skills for making dietary and physical activity changes vs Usual care	289; 74%; mean 52	Latino	PO: weight loss and HbA1c SO: fasting lipids, glucose, and insulin concentrations; BP; dietary assessment; physical activity measurements; and quality of life and depression scores.
52	Honduras and Mexico; private and public clinic, primary care practice, a diabetes specialty clinic, and community outreach; Patients with limited health insurance	RCT; 6 weeks	Monitoring focused: '—electronic home BP monitor —weekly automated monitoring and behaviour change calls vs Information only	181; 67.4%; 57.6 ± 0.8	not reported	PO: SBP SO: patients’ perceived general health, depressive symptoms, medication-related problems, and satisfaction with care.

(*Continued*)

**Table 1:** Continued

**Citation**	**Country; setting; SES definition**	**Design; intervention length; Follow up**	**Components of intervention (I) vs Control (C)**	**No. analysed; Female%; Age (mean,SD)**	**Race/ethnicity**	**Primary outcome (PO); secondary outcome (SO)**
44	US; A satellite community health center; Fair Oaks Clinic, 14 700-person, low-income, and largely Latino (73%) unincorporated neighborhood	RCT 12 months; 24 months	Lifestyle intervention: CM: —motivational interviewing—building self-management and goal setting skills —providing hands on cooking and physical activity demonstrations —fostering self-efficacy —leveraging group based social support —identifying community resources coordinating with primary care providers CHW + CM: —building broad skills for navigating an obesogenic environment —fostering family support —enhancing participant success in food negotiations —mapping out neighborhood walking routes —engaging participants in a modified Photovoice activity vs Usual care	207; 76.8%; 47.1 ± 11.1	Latino	PO: change in BMI SO: change in obesity-related cardiovascular risk factors at 6, 12, and 24 months. Obesity-related cardiovascular risk factors included: waist circumference, SBP, DBP, fasting blood glucose, HbA1C, total CHL, HDL, LDL, triglycerides, and C-reactive protein.
45	US; Primary care clinics; low-income African Americans and Latinos	Cohort; 4 weeks; 6 months	Education focused: —informatin sessions —provocative questioning —referrals as needed	73; not reported; not reported	57.5% non Hispanic black; 35.6% Hispanic	No PO or SO defined. Short term outcomes: changes in knowledge related to nutrition, diabetes management, monitoring blood glucose levels and avoiding diabetes complications. Long term outcomes: change in HbA1Cm, BP, fasting or random blood sugar levels, weight, height, BMI, LDL, type of diabetes, comorbidity, and manners in which diabetes was managed. Patient reported measures: changes in general health medication adherence, readiness to change eating habits, and readiness to change exercise habits.

(*Continued*)

**Table 1:** Continued

**Citation**	**Country; setting; SES definition**	**Design; intervention length; Follow up**	**Components of intervention (I) vs Control (C)**	**No. analysed; Female%; Age (mean,SD)**	**Race/ethnicity**	**Primary outcome (PO); secondary outcome (SO)**
46	US; Primary care practices; A federally designated medically underserved area (either of 2 federal designations: medically underserved area or health professional shortage area); a current Medicare beneficiary	RCT; 12 months; 5 year	Monitoring focused: —videoconferencing with nurse case managers —home glucose meter —access to clinical data—access to a special educational webpage vs Usual care	1665; I: 63.5% C: 62.1% I: 70.8 ± 6.5 C: 70.9 ± 6.8	Intervention: 15.3% African American (non Hispanic); 35.8% Hispanic; 48.2% White (non Hispanic); 0.7% other; Control: 14.5% African American (non Hispanic); 34.6% Hispanic; 50.6% White (non Hispanic); 0.2% other	PO: HbA1c, LDL, BP No SO defined.
47	US; Federally Qualified Community Health Center; A predominantly Latino, Mexican heritage, Spanishspeaking, immigrant population	RCT; 6 months	Behaviourall intervention: —co-location of the clinical team —warm hand-off from the medical provider to be a behavioural health provider —shared treatment plan —up to 4 integrated medical visits for management of diabetes, psychological and behavioural factors —care coordination —6 culturally appropriate group health education classes vs Usual care	456 63.7%; 55.75 ± 9.82	Hispanic	PO: change in HbA1c SO: change in lipids and BP

(*Continued*)

**Table 1:** Continued

**Citation**	**Country; setting; SES definition**	**Design; intervention length; Follow up**	**Components of intervention (I) vs Control (C)**	**No. analysed; Female%; Age (mean,SD)**	**Race/ethnicity**	**Primary outcome (PO); secondary outcome (SO)**
48	US; Safety net primary care clinics; Individuals of low socioeconomic status, racial/ethnic minority, and/or limited health literacy/English proficiency	2×2 RCT; 18 months	Behavioural intervention: CKD registry: team based CKD management CBT: CKD registry+SMS: education materials, telephone self-management programme, telephone based health coaching vs Usual care	137; 51.8% 55 ± 12.2	42.3% Black or African American; 36.5% Hispanic; 14.6% Asian/pacific islander; 6.6% Caucasian/white	PO: change in SBP SO: change in the proportion of patients with BP control and albuminuria severity; changes in patient-reported self-efficacy of chronic disease management, communication with providers, medication adherence, quality of life, and awareness of CKD
49	US; Non-profit community clinic; Uninsured minorities who earned < 150% of the federal poverty level	RCT (phase 1, 6 months); Cohort (phase 2, 6 months)	Education focused: —monthly group visits —weekly community health workers mobile health contract —additional bimonthly CHW contract vs Usual care	37 in phase 1; 54.1% I: 52.5 ± 7.8 C: 57.7 ± 9.2	Latino	PO: HbA1c change for phase II participants SO: viewing potential differences between the research versus clinic team by comparing the research team- led arm (phase I intervention) to the clinic team- lead arm (phase II)
51	Italy; Hospital; An urban peripheral quarter of Milan, Italy, composed of 4462 residents of any age at the time of study initiation, 43.6% were immigrants	Cohort; 12 months	Education focused: —personalised information —motivational interviewing —shared decision-making —use of local resources for a healthy lifestyle	369; 58.0%; 52.0 ± 7.5	not reported	PO: program uptake SO: sociodemographic determinants of the program uptake, prevalence of CV risk factors and conditions among adopters, awareness of traditional CV risk factors, accuracy of CV risk perception, use of professional resources, retention in the program and changes in lifestyle, in individual risk factors and in a pre-defined index of global risk change.

RCT: randomised controlled trial; BMI: body mass index

BP: blood pressure; SBP: systolic blood pressure; DBP: diastolic blood pressure

CVD: cardiovascular disease; CKD: chronic kidney disease

HbA1C: glycosylated haemoglobin

CHL: cholesterol; LDL: low-density lipoprotein cholesterol; HDL: high-density lipoprotein cholesterol

ED: emergency department

FAFQ: The Fat and Fibre Questionnaire; FVS: All Day Fruit and Vegetable Screener; CHAMPS: Community Healthy Activities Model Program for Seniors

### Risk of bias

Quality assessment was completed for each included study. Of 13 RCTs, 10^33^^,^^35^^,^^39^^,^^41^^,^^43^^,^^44^^,^^47^^,^^48^^,^^50^^,^^52^ were rated as low risk of bias and three^40^^,^^46^^,^^49^ as medium risk of bias largely due to the inadequate description of the blinding process, the precision of the estimated effect and potential harms and costs. Two cohort studies^38^^,^^45^ were rated as low risk of bias, two^37^^,^^42^ medium risk of bias and one^36^ high risk of bias.

### Intervention and reported effects

Of 10 studies that reported the change in weight, six observed a decrease and three observed no difference. The conclusion could not be drawn in one study where no sufficient information was reported.^36^ Six of the 10 studies that reported the change in BMI observed a decrease, three observed no difference and one comprised insufficient information^36^ to be asssessed. The majority of the studies that reported BP, total cholesterol, LDL and HDL observed no difference at follow up. A reduction was observed in all studies that reported HbA1C except for no difference in one study.^44^ Reported outcomes are presented in Table 2.

**Table 2:** Effect direction plot for reported outcomes of included studies (*n* = 19)

**Intervention type**	**Citation**	**Weight**	**BMI**	**SBP**	**DBP**	**HbA1C**	**Total CHL**	**LDL**	**HDL**
**Behavioural**	35 ^*^ ^,^^a^	▲	▲						
	50 ^*^ ^*^ ^,^^a^	▲	▲	◄►	◄►				
	47 ^*^ ^,^^a^			◄►	◄►	▲(%)	◄►	◄►	◄►
	48 ^*^ ^,^^a^			◄►	◄►				
**Lifestyle**	36 ^c^	cannot tell	cannot tell	cannot tell				cannot tell	
	37 ^b^	▲	▲	◄►	◄►				
	33 ^*^ ^,^^a^	▲		◄►	◄►		◄►	◄►	▼
	40 ^*^ ^*^ ^,^^b^		◄►(12w) ▲(12 m)	◄►	◄►		▼(12w) ◄►(12 m)	▼(12w) ◄►(12 m)	
	39 ^*^ ^*^ ^,^^a^		◄►	◄►	◄►		◄►	◄►	
	43 ^*^ ^,^^a^	▲	▲			▲			
	44 ^*^ ^,^^a^	◄►	◄►	◄►	◄►	◄►	◄►	◄►	◄►
**Education based**	38 ^a^			▲	▲	▲	▲	▲	◄►
	42 ^b^	◄►		◄►	◄►		◄►		
	45 ^a^	◄►		◄►	◄►	▲ (%)		◄►	
	49 ^*^ ^,^^b^	▲(phase1) ▲(phase2)	→(phase1) ▲(phase2)	→(phase1) → (phase2)	→(phase1) ▲(phase2)	▲(%,phase1) ▲(%,phase2)			
	51 ^a^		▲	▲	▲			▲	▼
**Medication based**	41 ^*^ ^*^ ^a^			◄►	◄►				
**Monitoring based**	52 ^*^ ^*^ ^,^^a^			▲	▲				
	46 ^*^ ^,^^b^			▲(low income group)		▲(%; low income group)		◄►	

▲: positive health impact;▼: negative health impact; ◄►: no change

^*^For RCTs, this relates to the reported difference between intervention and control arms at follow up; For cohorts, this relates to the reported difference between baseline and follow up.

^*^^*^RCTs where usual care was not used as control arm

^a^ = low risk of bias; ^b^ = some concerns; ^c^ = high risk of bias

#### Behavioural interventions

Four studies^35^^,^^47^^,^^48^^,^^50^ tested behavioural interventions for the management of weight, diabetes, hypertension and CKD. Two tested a behavioural weight management programme comprising cognitive–behavioural interventions, self-monitoring, dietary and physical activity advice and skills training. One compared with the usual care in populations with low literacy and limited access to health services,^35^ and the other compared with group-based advice and support on diet and physical activity from the practice nurse with people living with high levels of social deprivation.^50^ Both studies reported lowered weight (−1.07, 95% confidence interval (CI) −1.94 to −0.22; −1.9, 95% CI –3.7 to −0.1) and BMI (0.41, 95% CI -0.73 to −0.09; −0.7, 95% CI -1.3 to 0.0) but no differences were identified in blood pressure.

One study compared usual care in people in low income, Spanish-speaking Latinos with type 2 diabetes with the special intervention comprising integrated medical and behavioural co-located visits, group-based diabetes self-management education sessions and care coordination.^47^ HbA1C was lowered (−0.32, 95%CI −0.49 to −0.15), but cholesterol remained unchanged.

In a 2×2 study,^48^ patients with CKD from the safety-net primary care clinics received access to the CKD registry with feedback or a self-management programme or both. However no difference was observed in SBP in any of the intervention groups.

#### Lifestyle interventions

Five RCTs^33^^,^^39^^,^^40^^,^^43^^,^^44^ and two cohort^36^^,^^37^ studies investigated the effect of lifestyle interventions comprising physical activity, dietary intake support, education and skill building on metabolic syndrome,^36^ CVD^37^^,^^39^^,^^40^ and weight management.^33^^,^^43^^,^^44^ Results were unable to be synthesised in one^36^ of the cohort studies rated a high risk of bias due to the lack of 95% CI or *P*-value reported. In the other cohort study,^37^ participants with lower socio-economic status and less access to health care were provided with access to physical activity, dietary intake activity and heart-healthy education. Participants at the follow up experienced a weight reduction (*P* < 0.001, neither mean difference (MD) nor 95% CI reported) and BMI (*P* < 0.001), but their systolic and diastolic BP remained unchanged.

Three of the five RCTs reported the effects on weight at follow up compared with the usual care in low income populations. Two studies^33^^,^^43^ reported a greater reduction (−4.51 kg, 95% CI −6.01 to −3.02; −2.5 lb, 95% CI −4.25 to −0.75) and one^44^ reported weight as unchanged.

Four^39^^,^^40^^,^^43^^,^^44^ RCTs reported the effects on BMI at follow up compared with CVD risk screening plus education in women who were un-or underinsured in two studies^39^^,^^40^ and with usual care in low income populations in another two studies.^43^^,^^44^ A lowered BMI (*P* = 0.03, neither MD nor 95%CI reported; −0.46, 95%CI −0.76 to −0.14) at follow up was observed in two studies^40^^,^^43^ which remained unchanged in the other two studies.^39^^,^^44^

Studies^33^^,^^39^^,^^40^^,^^44^ which investigated the effects on both SBP and DBP reported no difference in BP between the intervention and the control at follow up.

Two RCTs reported the effects on HbA1C in low income populations^43^^,^^44^. Compared with usual care, a reduction (−0.07, 95%CI −0.10 to −0.04) was observed in one study^43^ whereas no difference in the other study.^44^

Four^39^^,^^40^^,^^43^^,^^44^ studies reported the effects on cholesterol in women who were un-or underinsured^39^^,^^40^ or low income populations.^33^^,^^44^ No differences were observed in total cholesterol and LDL at follow up. There was likely increased total cholesterol (*P* = 0.02, neither MD nor 95%CI reported) and LDL (*P* < 0.01, neither MD nor 95%CI reported) at 12 weeks, but they remained unchanged at 12 months.^40^ An increased HDL (4.6, 95%CI 2.9 to 6.3) was reported in one study,^33^ but it was unchanged in the other study.^44^

#### Education-based interventions

Five studies^38^^,^^42^^,^^45^^,^^49^^,^^51^ including four cohorts^38^^,^^42^^,^^45^^,^^51^ and one RCT,^49^ investigated education-based interventions combining information sessions, coaching sessions and motivational interviewing for lifestyle recommendations. Particpants included people with diabetes,^38^^,^^45^^,^^49^ CVD^42^^,^^51^ and hypertension, and hyperlipidemia^38^ and who were identified as either low income,^38^^,^^45^^,^^49^ older women with histories of substance abuse and homelessness^42^ and ethnic minorities.^51^

Of the three studies^42^^,^^45^^,^^49^ that reported the effects of the intervention on participants’ weight change, one^49^ observed a greater reduction in pounds (lbs) (*P* = 0.044, neither MD nor 95%CI reported) compared to the usual care at 6 months and the other two studies^42^^,^^45^ identified no difference.

BMI decreased (−0.3, 95%CI −0.2 to −0.5) in one cohort study^51^ but it was unchanged in an RCT compared with usual care.^49^

BP was reported in all five studies, both SBP (*P* < 0.001, neither MD nor 95%CI reported^38^; −7.2, 95%CI −5.6 to −8.8^51^) and DBP (*P* < 0.001, neither MD nor 95%CI reported^38^; −4.3, 95%CI −3.4 to −5.2^51^) were lowered in two cohort studies, but they remained unchanged in the other three studies.^42^^,^^45^^,^^49^

An improvement in HbA1C was observed in all three studies that reported the impact: *P* < 0.001^38^; *P* = 0.007^45^; *P* = 0.016^49^ (neither MD nor 95%CI were reported in these studies).

The outcome of cholesterol varied across studies that reported total cholesterol, LDL and HDL. Total cholesterol was lowered (*P* < 0.001, neither MD nor 95%CI reported) in one study^38^ but unchanged in the other study.^42^ LDL was lowered (*P* = 0.04, neither MD nor 95%CI reported^38^; −7.2, 95%CI −4.7 to −9.7^51^) in two studies but unchanged in one study.^45^ HDL was increased (1.2, 95%CI 2.1 to 0.3) in one study but unchanged in the other study.^38^

#### Medication-based interventions

One RCT investigated the effects of medication-based interventions on people living with hypertension who had low income.^41^ It compared interventions comprising an algorithm of antihypertensive medication adjustments, higher antihypertensive medication if needed and weekly telephone health coaches with patients receiving all but without antihypertensive medication.^41^ Both groups had a reduction in SBP and DBP, but no difference between them. However, when data from the two groups were combined, SBP significantly decreased by 21.8 mmHg between baseline and 6 months (*P* < 0.001). This suggested that health coaching itself was associated with improved blood pressure.

#### Monitoring-based interventions

Two RCTs studied the effects of monitor of BP and glucose amongst people with limited health insurance^52^ and those from underserved areas.^46^ A greater reduction in SBP was found in both those who had low literacy with high information needs (−8.8, 95%CI −14.2 to −3.4),^52^ and those who had the lowest income level (−4.23, *P* = 0.019, no 95%CI reported).^46^ A lower HbA1C but unchanged LDL was reported (−0.5, *P* ≤ 001, no 95%CI reported) in those with the lowest income level.^46^

## Discussion

This review synthesised interventions targeting CVD risks and outcomes and the effects reported on clinical outcomes for socioeconomically disadvantaged populations. A total of 19 studies with mixed quality of evidence were included resulting in five types of multifaceted interventions that were based on behavioural change, lifestyle, education, medication and monitoring. Mixed effects were reported for clinical measures that include weight, BMI, BP, HbA1C and cholesterol with inadequate evidence to determine whether any of the intervention types are effective in optimising lipids management for socioeconomically disadvantaged populations.

There was a limited definition of socioeconomically disadvantaged populations observed across the included studies. Although this review was set up to include populations with low levels of income, education, social class, deprivation, poverty, or an area-based proxy for deprivation, studies included commonly recruited participants according to their income, ethnicity or health insurance. None related to educational attainment or employment status, which are also important makers of socioeconomic status associated with CVD outcomes, particularly in high-income countries. There is a strong association between education and health literacy, which is likely found to be low in those who experience increased all cause mortality^53^ or with low or no compliance with their medications.^54^ In another study, the unemployed population showed an increased risk of CVD events than the retired cohort after controlling for demographic factors,^55^ indicating that job loss could lead to the negative effects of unemployment. Given the dynamic changes between these factors and CVD risks in one’s life, multiple markers of socioeconomic status should be used in research and practice in predicting CVD risks or outcomes.

None of the included studies measured mental health except one which reported the unchanged mental health status, as part of the 12-item Short Form Health Survey, being unchanged compared with usual care.^48^ There is evidence increasingly suggesting that psychological factors affect socioeconomically disadvantaged populations and their CVD outcomes. Individuals with low income who experienced stress and depression reported over 45 and 30% higher risk of developing CVD and all cause mortality respectively after controlling for demographic, clinical and behavioural factors.^56^ Similarly, a higher risk of CHD mortality was also reported in another study amongst those with both low socioeconomic status and psychological distress.^57^ The inequalities in risks may highlight inadequate resources to address psychological distress events and related health behaviours including physical inactivity and smoking, at both patient and practice levels. Future interventions and research should also evaluate the impact of interventions on mental health wellbeing targeting CVD in socioeconomically disadvantaged populations.

This review showed the inconsistent impact of multifaceted interventions on lipid management for socioeconomically disadvantaged populations, given the wide variety of settings, intervention components, approaches and targeted populations. Although interventions for CVD in general populations seem effective, difficulty in concluding the effectiveness of any interventions for vulnerable groups has been reported by the previous literature.^58^ This issue with large variety has also been suggested in the past literature as a barrier to determining the effectiveness of the interventions for socioeconomically disadvantaged groups,^59^ however it is required and almost necessary to address extra care needs of these specific patient groups.^60^ Reviews with less broad eligibility focusing on more specific cohorts with subgroups analysis may be valuable to detect breakdown effects. It is also worth noting that most of the interventions examined in this review were adapted to specific research settings requiring extra resources, for example, materials being translated and contents being simplified for readability. This raises a question on the sustainability of scale up implementation within health systems. Future research using health economic evaluation is needed to confirm the cost-effectiveness.

Although this review had no limitation on study design when searching records in the databases, only studies with RCTs and cohort designs were included. Future studies are needed using qualitative or mixed methods to reflect lived experience and describe barriers and challenges in intervention delivery and implementation in real world settings.

### Limitations

This review was limited by the fact that only studies undertaken in OECD countries published in English were included. This was to ensure the similarity of healthcare systems and socioeconomic and demographic structure, therefore the findings may be less generalisable for socioeconomically disadvantaged populations in low- or middle-income countries, where research reporting CVD in socioeconomically disadvantaged populations is limited often with conflicting results.^61^^,^^62^ In addition, potential theoretical bias may exist given the intervention was delivered or facilitated by either clinicians or researchers which may result in a placebo effect.

## Conclusion

This review synthesised 19 studies presenting five types of intervention type focusing on behaviours, lifestyle, education, medication and monitoring. Definition of disadvantaged socioeconomic populations was inconsistently used to describe mainly relating to racial/ethnic minorities, low income and limited or no health insurance. There is inadequate evidence to determine whether any of the intervention types are effective in optimising lipids management for socioeconomically disadvantaged populations, due to a large variety of settings, participants and intervention components although they are considered necessary to address the complex health needs of socioeconomically disadvantaged populations in practice. Future research is needed with multi-factor defined populations using mixed evidence using real world evaluation and lived experience combined with health economic evaluation, on both mental and physical health outcomes.

## Author contributions

YF led the study design contributed by GT and KT. YF drafted the manuscript revised by GY, NW and KT. All contributed to data analysis, revised the draft manuscript and approved the final version.

## Credit author statement

Yu Fu (Conceptualization, Formal analysis, Methodology, Resources, Supervision, Visualization, Writing—original draft, Writing—review & editing), Ge Yu (Formal analysis, Methodology), Naswa Maulana (Formal analysis, Investigation, Project administration, Resources, Writing—review & editing), and Katie Thomson (Formal analysis, Methodology, Resources, Writing—review & editing)

## Conflicts of interest statement

The authors have no potential conflicts of interest.

## Funding

This work is supported by the National Institute of Health Research (NIHR) [Applied Research Collaboration North East and North Cumbria (NIHR200173)]. The views expressed are those of the author(s) and not necessarily those of the NIHR or the Department of Health and Social Care.

## Data availability

No new data were generated or analysed in support of this review.

## Patient consent

Not required.

## Ethical approval

Not required.
